# Case of Encysted Abscess in the Spinal Cord

**Published:** 1831-01-01

**Authors:** 


### MISCELLANIES.

LXX.

### I. CASE OF ENCYSTED ABSCESS IN THE CENTRE OF THE SPINAL CORD. By J. HART, Esq.

THIS case is reported in the last volume of the Dublin Hospital Reports. It deserves a short notice, more as a matter of curiosity indeed, than of practical utility.

In the Summer of 1828, Mr. Hart assisted at the post-mortem examination of a child, at which Mr. Crampton was present. "The child had been for about a month suffering from irritation of the bowels, which the parents supposed to be owing to dentition, when a lateral curvature of the lumbar portion of the spine to the right side was taken notice of, accompanied with spasmodic twitching of the right leg and thigh. Soon after this occurrence, the child was affected with convulsions, which became more frequent, and were shortly accompanied with well marked symptoms of hydrocephalus, which, notwithstanding very active treatment, proved rapidly fatal. On examination of the body the following appearances were found. The head was enlarged, the sagittal and coronal sutures open, the pia mater every where red from increased vascularity; there was a considerable effusion of straw-coloured serum on the surface of the brain, in the ventricles, which were distended, and in the spinal canal. In fact, there were the ordinary appearances so well known to present themselves after death from hydrocephalus.

We next examined the spinal canal, when we found a globular sac, of about the size of a hazel nut, filled with an aqueous fluid, which pressed on the nerves of the cauda equina of the right side, accounting for the spasmodic twitchings of the muscles of the limb.

Observing that the spinal cord appeared of unusual thickness, an incision was made into it, from which some *thick purulent matter* flowed out; on further examination, we found that this matter was contained in a cyst, which occupied the very centre of the spinal cord: this cyst was of an oblong form, extending from the first to the twelfth of the dorsal vertebræ, and was terminated by an obtuse extremity both above and below. The diameter of this tubular cyst was about four lines, its parietes were a line in thickness, and so solid as not to collapse when the contained fluid was discharged; so completely did it occupy the centre of the spinal cord, that the medullary substance formed a tube of equal thickness on every side of it. The absence of paralysis in this case is remarkable."

The preparation is deposited in the Park Street Museum, and a drawing of a secton of the cord accompanies the case.

### II. OBSTINATE PSORIASIS SUCCESSFULLY TREATED.

DR. WEST has related a case of this kind in the last volume of the Dublin Hospital reports, which is worth recording.

*Case.* A young man, of robust constitution, was attacked by psoriasis, which first appeared on the lower part of the back, then extended to the thighs and legs, and at last covered the whole body, with the exception of the face and hands. Plummer's pill and a mixture of the tar, citrine, and sulphur ointments were prescribed, then vapour and sulphur baths, and the internal exhibition of the liquor arsenicalis; in short, a variety of treatment was employed without effect. Having allowed a short interval of repose from all treatment, in order that the patient might regain his strength, he was put on the following plan. He was confined to bed, placed on a cooling and scanty vegetable diet, bled several times, and his bowels were kept open by a purgative electuary consisting of cream of tartar and electuary of scammony. The good effects of the treatment soon became apparent; the redness diminished rapidly, the formation of scales decreased in the same proportion, and in less than five weeks he was almost freed from the disease. There has been no relapse, nor is there any mark or discolouration of the skin.

### III. SINGLETON'S GOLDEN OINTMENT, AND AN IMPROVED RED PRECIPITATE OINTMENT.

MR. THOMAS CLARK has published in No. XII. of the Glasgow Journal an analysis of Singleton's Golden Ointment, which differs considerably from that of former chemists. Mr. Gray, Mr. Rennie, and Dr. Paris, had stated it to consist of lard and orpiment, sulphuret of arsenic. From experiments, for the details of which we refer to our contemporary, Mr. Clark comes to the conclusion that an ounce of the ointment consists of one drachm of the red precipitate, and seven drachms of butter. The said ounce is sold at *one guinea*, exclusive of the duty, and costs just *one penny;* a good illustration, as Mr. C. remarks, of the modern transmutation of metals. After some sprightly remarks on this and the red precipitate ointment of the Pharmacopœia, Mr. C. proposes a new formula for the latter. Here it is.

Red precipitate, prepared by nitric acid; good yellow wax, of each a drachm; prepared lard, an ounce.

Rub the red precipitate till it become orange. Then mix it with a little of the lard. Mix also the remainder of the lard with the wax, and melt them together. When the latter mixture is removed from the fire and has begun to harden, add to it the former mixture. Stir the whole together till it cool.

IV. MEDICAL BOTANY. By J. STEVENSON, M.D. and JAMES MORSS CHURCHILL, F.L.S. &c.

WE have much pleasure in informing our readers that this valuable work is now completed. On the necessity of the study of medical botany we need say nothing; common sense points it out, and, what is yet more likely to stimulate the lagging industry of the young aspirant to Æsculapian fame, the corporate bodies require it. Of the modern works upon the subject we believe that the present is by far the best. We have, on a former occasion, pointed out the general plan adopted by the authors, and we need not at present go over the ground again. Neither need we pretend to give a formal notice of what is neither susceptible of review, nor fit material for analysis. Such works do not come within the scope of a periodical journal, and we must therefore content ourselves with expressing our approbation of the manner in which it has been executed and the objects for which it was undertaken. It will form a valuable acquisition to every medical student, and an useful and elegant addition to every medical library.

---

